# Non-Learning BIM-Guided Weld Seam Localization from Partial Point Clouds for Robotic Welding of Steel Structures

**DOI:** 10.3390/s26216934

**Published:** 2026-11-02

**Authors:** Jiwon Jeong, Seungjun Ahn

**Affiliations:** 1 Department of Civil Engineering, Hongik University, Seoul 04066, Republic of Korea; xxx03018@g.hongik.ac.kr; 2 Department of Civil and Environmental Engineering, Hongik University, Seoul 04066, Republic of Korea

**Keywords:** weld seam, point-cloud registration, partial point cloud, non-learning, BIM, robotic welding, welding automation, steel structure, construction site

## Abstract

Restricted viewpoints on construction sites often yield partial point clouds in which weld seams are partly or entirely occluded. This study proposes a non-learning BIM-guided method that localizes BIM-defined nominal weld seams by registering a known H-section beam–column joint model to the visible flange and web surfaces. Given an isolated point cloud and the acquisition side, finite analytic member surfaces and a BIM-derived Hesse atlas support multiple admissible initial poses. Each pose is refined using the same multiscale robust point-to-surface objective, and a deterministic terminal criterion selects the pose used to transfer nominal seam paths. Neither seam labels nor reconstruction of occluded geometry is required. On 402 synthetic observations, median seam RMSE, ADD-RMS, and relative rotation error were 1.110 mm, 1.249 mm, and 0.067 degrees, respectively; 89.05% had seam RMSE within 10 mm. On 180 laboratory observations, median point-to-surface distance and 5 mm attachment were 2.137 mm and 82.04%. Median warm processing times on a matched panel were 2.52 s and 3.18 s for synthetic and laboratory inputs, respectively. These findings support the applicability of the proposed method to nominal weld-seam localization under restricted partial-view conditions relevant to construction-site welding.

## 1. Introduction

Automating welding operations on construction sites can reduce worker exposure to hazardous tasks, reliance on highly skilled manual labor, and process variability in steel construction. BIM-driven construction robots and robotic welding systems have demonstrated potential for safer and more reliable field operations [1,2]. Transferring welding automation from a controlled manufacturing cell to an erected steel structure, however, introduces a distinct perception challenge before the intended weld location can be used by an automated system. In manufacturing, the relative positions of the workpiece, fixture, robot, and sensor can generally be maintained in stable, repeatable spatial configurations, allowing the viewpoint to be selected for favorable seam visibility. On a construction site, the joint is embedded within a larger structural assembly, while adjacent members, temporary facilities, access routes, and the robotic platform restrict sensor placement. These constraints are particularly important in elevated work, where viewpoint selection cannot be governed solely by visibility. Consequently, the joint may be observed from an oblique or irregular direction, producing a partial point cloud in which substantial portions of the members are absent and the intended seam is partly or fully occluded. A construction-oriented perception method must therefore infer the seam location by aligning the joint model with the member surfaces that remain visible. Viewpoint-control research similarly indicates that the sensor viewpoint cannot always be chosen solely to maximize seam visibility [2].

Existing weld-seam perception studies primarily extract observable grooves, edges, intersections, or seam-bearing regions from images, laser measurements, and three-dimensional point clouds [3,4,5]. Point-cloud registration research has also developed global and low-overlap methods for estimating rigid transformations from incomplete observations [6,7,8]. Although learning-based approaches can extract discriminative features from partial point clouds, their deployment depends on training data that adequately represent the relevant joint geometries and sensing conditions [6]. Establishing such coverage can be difficult when joint dimensions, connection details, and sensing conditions vary, particularly when the nominal seam is not directly visible. Also, direct seam extraction requires recognizable seam evidence, whereas general registration seeks agreement between observed and reference geometry. In H-section joints, repeated, nearly parallel flange and web surfaces can support several geometrically similar placements while transferring a BIM-defined nominal seam to different locations. A remaining challenge is therefore to localize such a seam from a restricted partial view when the seam itself may be absent, without learned weld-region segmentation or reconstruction of unobserved geometry.

This study proposes a non-learning BIM-guided method for this task. Here, non-learning means that the geometric representations and pose updates are computed directly from the supplied BIM and observation, without trained model parameters or annotated datasets. New member dimensions and nominal seam paths can therefore be incorporated by regenerating the BIM representations.

The method takes point clouds as input and targets beam–column joints composed of H-section members, which are widely used in steel construction [9]. Point clouds provide direct three-dimensional geometry while reducing dependence on appearance cues affected by color, texture, and illumination [10]. Specifically, the method takes a known BIM instance and an externally preprocessed, isolated partial-object point cloud acquired under approximately upright and joint-facing conditions, together with binary acquisition-side information. It constructs finite analytic flange–web surfaces from the BIM-derived geometry, generates multiple admissible initial poses, refines every pose using the same surface objective, selects the final pose, and transfers the nominal seam paths into the observation coordinate system. The output is a localized nominal BIM seam, not an estimated physical weld root, and the method does not reconstruct missing geometry. Evaluation used seven reference methods, 402 synthetic observations comprising 201 paired Raw and Noise-corrupted samples, and 180 laboratory observations of one physical assembly. This validation focused on strong-axis joints, and exact ground truth was used for synthetic evaluation, while laboratory results were assessed through surface compatibility.

The contributions of this study are twofold. First, it extends construction-oriented weld-seam localization to a condition in which the target nominal seam is not required to be directly represented in the input point cloud. Second, it enables the nominal seam to be localized from the visible member surfaces through a non-learning BIM-guided approach, without weld-region training labels or missing-shape reconstruction.

The remainder of this paper is organized as follows. Section 2 reviews weld-seam extraction and localization approaches and registration methods for limited-view partial point clouds. Section 3 presents the proposed method, Section 4 describes the experiments and results, Section 5 discusses the findings and limitations, and Section 6 concludes the study.

## 2. Related Work

This study draws on two bodies of work: weld-seam localization and the registration of limited-view partial point clouds. Weld-seam localization identifies task-relevant seam geometry from sensor observations or digital reference models, whereas partial point-cloud registration estimates the rigid transformation relating an incomplete observation to known geometry. Section 2.1 and Section 2.2 review these domains in turn, and Section 2.3 synthesizes the remaining gap between them to position the present study.

### 2.1. Vision- and Reference-Model-Based Weld-Seam Localization

Vision-based weld-seam localization has been investigated using stereo cameras, RGB-D sensors, laser sensing, and three-dimensional point clouds. Dinham and Fang [11] developed an eye-in-hand stereo-vision system for seam identification and localization, while Ahmed et al. [3] extracted edges and corners from unorganized point clouds to construct welding seams. Peng et al. [12] similarly detected V-shaped grooves from RGB-D point clouds. These studies represent a geometry-driven approach in which seam positions are derived from observable edges, grooves, corners, or surface intersections.

Later studies incorporated multiple observations, viewpoint adjustment, learned features, and reference geometry. Kim et al. [13] registered seam points extracted from several RGB-D views to represent multiple and partially occluded seams. Liu et al. [14] detected semantic line features using deep learning, whereas Takubo et al. [4] refined welding-line detection by remeasuring the workpiece from optimized sensor positions. Wei et al. [5] combined coarse seam-region detection in RGB images with fine edge extraction from point clouds. Reference-model-based approaches have also aligned measured workpieces with CAD- or BIM-derived geometry: Y. Li et al. [15] employed an FPFH–RANSAC–ICP pipeline together with segmentation and geometric feature extraction, and T. Li et al. [16] used BIM-derived weld features and axis-direction information to register visual measurements with a workpiece model.

### 2.2. Registration of Limited-View and Low-Overlap Partial Point Clouds

Rigid point-cloud registration estimates the rotation and translation between two geometric representations. The iterative closest point method introduced by Besl and McKay iteratively updates point correspondences and rigid transformations, while fast point feature histograms provide local geometric descriptors for correspondence-based initial alignment [17,18]. Global methods have subsequently been developed for less favorable initial conditions. Super4PCS searches for congruent four-point configurations, fast global registration jointly optimizes a robust objective over feature correspondences, and TEASER estimates rigid transformations under high correspondence-outlier rates using truncated least-squares formulations [7,8,19].

More recent learning-based methods have focused on correspondence scarcity under partial and low-overlap observations. PREDATOR employs overlap-aware attention to identify points that are both geometrically informative and likely to belong to the shared region of two point clouds [6]. GeoTransformer learns transformation-invariant geometric representations from pairwise distances and angular relationships, enabling correspondence estimation under low-overlap conditions without a subsequent RANSAC stage [20]. These studies illustrate the progression of partial registration from handcrafted descriptors and geometric consensus toward robust optimization and learned overlap-aware correspondence extraction.

### 2.3. Knowledge Gaps

The reviewed weld-seam localization methods generally derive seams from observable grooves, edges, intersections, semantic lines, or predicted seam regions. When one view is insufficient, studies combine multiple views, reposition the sensor, or use coarse image localization before point-cloud refinement. CAD- or BIM-based approaches add reference geometry but still rely on observable features, segmentation, reconstruction, or additional sensing to identify the target. They therefore do not directly address a single restricted-view partial point cloud in which the nominal seam is absent.

**Figure 1:**
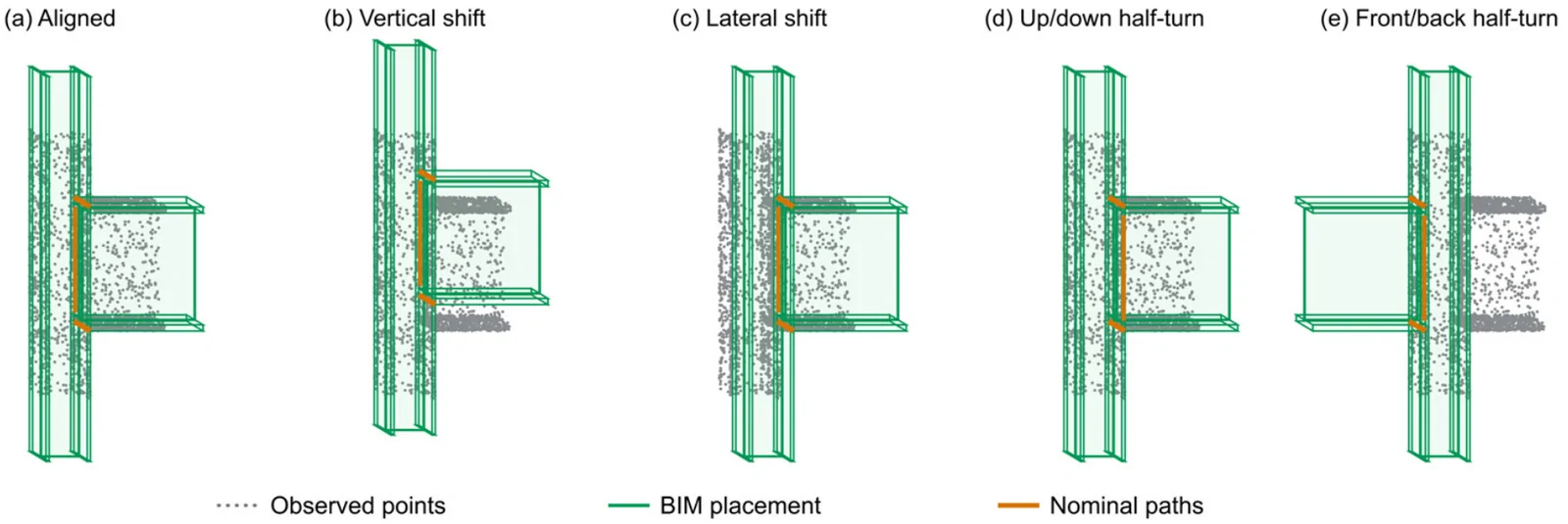
Repeated-surface ambiguity in a beam–column assembly. The same gray observed point cloud is repeated at the same position and viewing angle; green lines show alternative BIM placements, and orange marks show nominal paths. Besides the aligned placement, the examples illustrate vertical and lateral displacement and half-turn alternatives. These conceptual alternatives illustrate possible ambiguity.

Partial and low-overlap registration methods provide a basis for pose estimation from incomplete geometry, but their primary objective is surface alignment rather than task-specific seam placement. Feature correspondences, congruent sets, robust estimators, and overlap-aware representations seek a transformation that makes the observed and reference geometries mutually consistent. For an H-section joint, however, repeated and nearly parallel flange and web surfaces may permit alternative poses with similar surface compatibility while transferring the BIM-defined seam to different locations. Consequently, satisfactory surface registration does not necessarily ensure accurate nominal-seam localization. Figure 1 illustrates how repeated member surfaces can support alternative BIM placements with different nominal-seam locations. Table 1 summarizes the sensing conditions, seam-visibility requirements, reference models, learning requirements, outputs, and validation settings of the reviewed approaches.

To address this gap, the proposed method localizes potentially unobserved nominal seams from a restricted-view partial-object point cloud using known BIM-derived joint geometry. Its distinguishing feature is an observation-conditioned multi-pose initialization followed by equal finite-surface refinement and nominal-seam transfer.

## 3. Method

### 3.1. Problem Formulation and Framework Overview

Given a known BIM-derived beam–column joint and an externally preprocessed partial point cloud X, the proposed non-learning method estimates the rigid BIM-to-observation pose T = (R,t) in SE(3) and transfers every active BIM-defined nominal weld-seam path to the observation frame. A deterministic sample drawn once without replacement forms nested point sets $X_{C}$ ⊆ $X_{R}$ ⊆ X. The coarse subset $X_{C}$ supplies local geometric measurements for initialization, whereas every candidate trajectory and final-pose comparison uses the same refinement subset $X_{R}$. These subsets are two processing resolutions of one observation; they are not independent observations, and no pose-dependent resampling is performed.

The BIM input may define any nonempty subset of three nominal seam classes: the upper flange, lower flange, and vertical web joint. Let $C_{B}$ denote the active BIM-frame seam set. Once the terminal pose $\hat{T}$ has been selected, the localized observation-frame seam set is

(1) $${\hat{C}}_{O}=\hat{T}\left(C_{B}\right)$$

**Figure 2:**
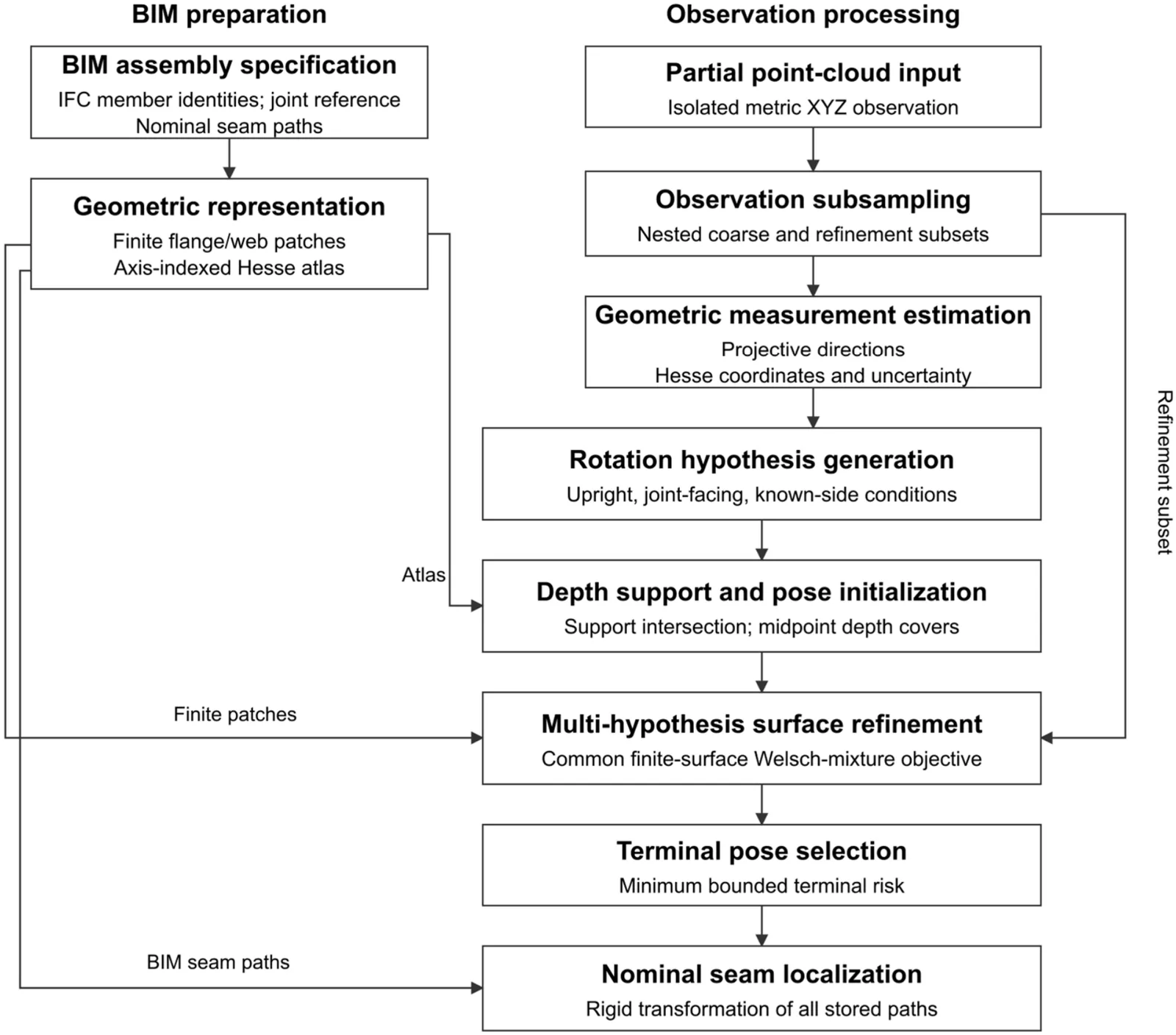
Processing flow from an isolated partial point cloud and BIM-derived geometric assets to localized nominal seam paths. Acquisition conditions constrain initialization only. Every initial pose undergoes the same finite-surface refinement, followed by terminal-risk selection; no initial pose is selected by predicted final quality.

The workflow shown in Figure 2 separates three roles: BIM representation, observation-conditioned initialization, and equal local refinement followed by deterministic terminal selection. The sensor is assumed to be approximately upright and directed toward the joint neighborhood. A binary acquisition-side sign is supplied only to initialization; exact camera pose, ground-truth registration, observed member labels, RGB information, and robot kinematics are not supplied.

### 3.2. BIM-Derived Geometric Representation

The geometric preparation process begins with an H-section beam–column assembly whose members are identified by standard IFC classes, including IfcBeam and IfcColumn. Two complementary representations are prepared in its common BIM coordinate frame: bounded flange and web patches for local surface fitting, and an axis-indexed Hesse-coordinate atlas for proposing initial placements. To construct the finite patches, beam and column triangles are processed separately to recover the longitudinal and cross-section directions. Signed flange and web levels determine the section dimensions, and longitudinal bounds determine the member lengths. These quantities define the fitting geometry once, before observations are processed. The K finite analytic patches are written as

(2a) $$R_{k}\left(\alpha ,\beta \right)=o_{k}+\alpha u_{k}+\beta v_{k},$$

(2b) $$\alpha_{k,min}\leq \alpha \leq \alpha_{k,max},\;\;\beta_{k,min}\leq \beta \leq \beta_{k,max}$$

where $o_{k}$ is the rectangle origin, $u_{k}$ and $v_{k}$ are in-plane unit directions, and the scalar bounds define the finite extent of the rectangle. Their union is

(2c) $$S_{B}=\overset{k=1}{\underset{K}{⋃}}R_{k}$$

The finite bounds preserve the member extents, allowing the closest point to lie on a face interior, an edge, or a corner. Distances to this union therefore account for boundaries that would be lost if the member faces were replaced by infinite supporting planes.

Using these nominal patches for fitting assumes that the manufactured beam and column faces are reasonably approximated by the BIM member geometry. Structural-steel fabrication and erection specifications control dimensional, straightness, alignment, and placement deviations [9,21], providing a domain basis for this approximation rather than a guarantee of exact agreement. Modest deviations enter as surface residuals; changed section dimensions or substantial deformation require an updated geometric model. Here, the analytic fitting primitives represent the beam and column flanges and webs, not plates, bolts, stiffeners, fillets, or weld details. Nominal seam paths are stored separately and transferred after pose estimation.

For initialization, the same BIM-derived mesh is reduced to a simpler Hesse-coordinate atlas. In the normalized plane form a ℓ^T^p = h [22], h is the signed surface coordinate along unit assembly axis a ℓ, denoted $a_{l}^{B}$ in Appendix A to make its BIM frame explicit. Each axis-aligned source triangle contributes its minimum and maximum vertex projections onto that axis; overlapping intervals are merged according to Equation (A9). The resulting atlas lists the one-dimensional coordinate supports at which aligned BIM surfaces occur.

Unlike the finite patches, the atlas omits each surface’s in-plane position and semantic identity. This reduced representation supports alternative initial translations without claiming to identify the correct placement. Every proposed pose is subsequently tested against the bounded patches during refinement. The two representations share the BIM coordinates and joint reference $c_{J}$, so the final pose also transfers the separately stored nominal seam paths without another coordinate conversion.

### 3.3. Observation-Conditioned Initial-Pose Set

Initialization constructs several admissible starting poses from one partial observation. It is not intended to predict which start will produce the best refined pose. On the coarse subset $X_{C}$, a 12-nearest-neighbor covariance estimate provides an unoriented local plane normal $n_{i}$ [23]. A small neighborhood supplies enough points for a stable plane estimate while limiting the tendency to mix adjacent faces; 12 is the fixed neighborhood size used here. The same neighborhood supplies an area proxy, directional confidence, and uncertainty in the normal and signed surface coordinate, as summarized in Appendix A. These quantities are computed once before pose generation and do not constitute semantic segmentation.

Because $n_{i}$ and −$n_{i}$ describe the same plane, the normal directions are treated projectively. Starting from the eigenvectors of their second projective moment, a local SO(3) update estimates an orthonormal frame Q by minimizing

(3a) $$F\left(Q\right)=\frac{1}{N_{C}}\sum_{i=1}^{N_{C}}\left[1-\underset{j\in \{1,2,3\}}{max}{\left(n_{i}^{T}q_{j}\right)}^{2}\right]$$

Here **q***_j_* is the *j*th column of **Q**. The loss is small when each observed normal lies near at least one frame axis, while the squared inner product removes the unknown normal sign. This construction is related to Manhattan-frame estimation [24], and the local rotation update follows standard SO(3) optimization [25]. The resulting stationary representative is denoted $Q_{O}$ in Appendix A.

One projective frame still permits axis permutations and sign changes. The method therefore retains the complete 24-state proper signed-permutation orbit. States inconsistent with the supplied acquisition side are removed, and gravity fixes the roll of each remaining viewing direction. These tests retain a set of admissible rotational initializations; they neither rank the rotations nor constrain subsequent refinement.

For each retained rotation, the uncertain observed surface coordinates are projected onto the three rotated BIM axes and compared with the corresponding Hesse-atlas intervals. Minkowski interval differences give the translations along each axis for which an observed level can coincide with a BIM level. Reliable local planar evidence contributes support, whereas uncertain evidence widens the admissible set rather than being treated as contradictory. Intersecting the three axis-wise supports gives the translation region associated with that rotation.

The joint-facing assumption parameterizes the initial translation using a single positive-depth variable. If the transformed BIM joint reference lies at depth *λ* on the observation optical axis $e_{z}$, the translation is

(3b) $$t_{r,\lambda }=\lambda e_{z}-R_{r}c_{J},\;\;\lambda >0,$$

Substituting Equation (3b) into the three coordinate supports gives allowed intervals of *λ*, which are intersected without filling disconnected gaps. Each connected interval is sampled by the union of midpoint covers with radii 60, 45, and 30 mm. Each radius bounds the distance to the nearest depth node and determines the subdivision count in Equation (A17). These values give maximum node spacings of 120, 90, and 60 mm, balancing centimeter-scale placement resolution against growth of the initial set. The factor-of-two range and intermediate 45 mm cover reduce dependence on one midpoint partition. These numerical settings are not a localization tolerance or guaranteed attraction-basin size; Section 4.4 assesses their robustness through complete-set scaling by 0.75 and 1.25. The resulting rotations and depths form

(3c) $$T_{0}={\left(T_{0}^{\left(m\right)}\right)}_{m=1}^{M_{0}},\;\;T_{0}^{\left(m\right)}=\left(R_{r\left(m\right)},\lambda_{m}e_{z}-R_{r\left(m\right)}c_{J}\right)$$

Every member of this ordered set is passed to refinement. No coarse surface score, top-k rule, non-maximum suppression, or predicted terminal quality is used to select among them. If the set is empty, the method returns an explicit no-pose outcome. Appendix A gives the compact definitions of the observation measure, Hesse-coordinate support, and depth materialization.

### 3.4. Robust Refinement, Pose Selection, and Nominal Seam Transfer

Each initial pose is refined independently with the same refinement subset $X_{R}$ and the same finite BIM-derived surface field. For T = (R,t), an observation point is first mapped into the BIM frame as

(4a) $$q_{i}\left(T\right)=R^{T}\left(x_{i}-t\right)$$

The mapped point is projected exactly onto the finite rectangle union $S_{B}$, and $d_{i}$(T) is its Euclidean distance to the closest finite face, edge, or corner. This retains the alternating closest-surface principle of ICP [17] but avoids replacing the BIM geometry with a sampled target point set. Distances and bandwidths use meters internally.

All trajectories minimize the same equally weighted Welsch mixture, where σ denotes a bandwidth:

(4b) $$J_{mix}\left(T\right)=\frac{1}{N_{R}}\sum_{i=1}^{N_{R}}\frac{1}{3}\sum_{\sigma \in \{15,\;7.5,\;5\}\;mm}\left[1-exp\left(-\frac{d_{i}{\left(T\right)}^{2}}{2\sigma^{2}}\right)\right]$$

The Welsch influence decreases continuously with residual magnitude, so a single narrow bandwidth can suppress initially displaced points that remain useful for alignment. The 15 mm component retains a broader response to these offsets, the 5 mm component emphasizes close surface agreement, and the intermediate 7.5 mm component links the two ranges. Their equal mixture retains capture and fine-alignment terms in one fixed objective rather than switching objectives between stages. Section 4.4 tests the contribution of this combined response by replacing it with a single 5 mm loss while retaining the same update stages. The corresponding IRLS weight and finite-feature Gauss–Newton model are given in Appendix B, following robust M-estimation [26] and robust registration [27].

At each accepted pose, the closest finite feature determines whether the local model uses the face normal alone or also the edge and corner directions. The local SE(3) action chart is re-anchored at the current pose [25]. The quadratic step is constrained by an RMS displacement limit over the unique rectangle corners, following the trust-region principle [28]. Limits of 15, 7.5, and 5 mm progressively reduce linearization error as alignment becomes more local, while remaining commensurate with the objective bandwidths. They bound individual updates, not the cumulative correction. Armijo backtracking accepts only objective-decreasing proposals [29].

After every initial pose has received the same refinement procedure, a single-scale 2 mm Welsch risk compares the terminal poses. Whereas the mixture retains a response to initial offsets during optimization, terminal comparison should distinguish smaller remaining offsets. The narrower 2 mm bandwidth provides this discrimination while keeping the contribution of unmatched points bounded. It is a continuous comparison scale, not an acceptance threshold or a sensor-accuracy specification. The effect of this separation is examined in Section 4.4 by applying 1, 2, and 5 mm risks to exactly the same refined poses, thereby varying the ordering scale without changing the optimization trajectories. The terminal risk is

(4c) $$J_{2}\left(T\right)=\frac{1}{N_{R}}\sum_{i=1}^{N_{R}}\left[1-exp\left(-\frac{d_{i}{\left(T\right)}^{2}}{2{\left(2\;mm\right)}^{2}}\right)\right]$$

The selected terminal pose is

(4d) $$\hat{m}=min\underset{1\leq m\leq M_{0}}{arg\;min}J_{2}\left(T_{F}^{\left(m\right)}\right),\;\;\hat{T}=T_{F}^{\left(\hat{m}\right)}$$

The minimum-index rule makes exact ties deterministic. Initialization provenance, acquisition-side information, and numerical stopping state do not enter this final ordering. The selected pose is applied to every active nominal seam path through Equation (1). The output is therefore a localized nominal BIM seam, not a reconstructed surface, fabrication-deviation estimate, or direct measurement of an as-built weld root. Appendix B summarizes the finite projection, IRLS model, action metric, and terminal selection. The complete localization procedure is summarized in Algorithm 1.
**Algorithm 1.** Non-learning BIM-guided nominal weld-seam localization**Input:**Preprocessed partial point cloud; finite BIM-derived surface field; BIM Hesse-coordinate interval atlas; joint reference; active nominal seam paths; binary acquisition-side sign**Output:**Selected BIM-to-observation pose and localized nominal seam paths, or an explicit no-pose outcome1:Construct the nested point sets $X_{C}$ ⊆ $X_{R}$ ⊆ X and compile the two BIM-derived geometric representations, joint reference, and active seam paths2:Estimate projective normals and their uncertainty from $X_{C},$ and obtain a locally refined orthonormal projective frame3:Expand the frame into its 24 proper signed states, apply side and gravity admissibility, and map each retained state to an upright rotation4:Initialize the deterministically indexed initial pose set $T_{0}$ as empty5:**for** each retained rotation $R_{r}$ **do**
6:  Compute all three supported coordinate sets and their common positive joint-depth interval set $\Lambda_{r}$
7:  **for** each connected interval [a, b] in $\Lambda_{r}$ **do**8:    Sample the 60, 45, and 30 mm midpoint covers and remove duplicate depths9:    Append every $(R_{r},$ $\lambda e_{z}$ $-\;R_{r}$ $c_{J}$) to $T_{0}$ in deterministic order10:  **end for**11:**end for**12:**if** $T_{0}$ is empty **then return** an explicit no-pose outcome13:Initialize the ordered refined-pose set $T_{F}$ with length $M_{0}$
14:**for**$\;m=1,\;\ldots ,\;M_{0}$ **do**15:  Refine ${T_{0}}^{\left(m\right)}$ with the common finite-surface Welsch objective, GN/IRLS model, action limits, and Armijo rule16:  Store the resulting terminal pose as ${T_{F}}^{\left(m\right)},$ preserving index m17:**end for**18:Set $\hat{m}\leftarrow min\;\underset{1\leq m\leq M_{0}}{arg\;min}\;J_{2}({T_{F}}^{\left(m\right)}),$ $\hat{T}\leftarrow {T_{F}}^{\left(\hat{m}\right)},$ and transform all active seam paths using $\hat{T}$
19:**return** $\hat{T}$ and the localized nominal seam paths

## 4. Experiments and Results

### 4.1. Experimental Design and Implementation

The evaluation comprised two complementary benchmarks. The synthetic benchmark, for which exact pose ground truth was available, compared nominal-seam localization and pose accuracy, whereas the laboratory benchmark evaluated point-to-surface compatibility and qualitative nominal-seam transfer under laboratory conditions without independent pose or seam ground truth.

The proposed method used at most 6000 observed points for initialization and refinement, drawn once without replacement with PCG64 seed 0; all points were retained when fewer were available. Local geometry was computed only for initialization. Each candidate received at most 64 iterations per refinement stage, with an Armijo coefficient of 10^−4^. The CPU/GPU implementation was evaluated on an Intel Core i9-10900X processor, 64 GB of system memory, and an NVIDIA RTX 3090 with 24 GB GPU memory.

Seven comparison methods were evaluated: centroid + ICP, FPFH–RANSAC + ICP, FGR + ICP, Super4PCS + ICP, TEASER++ + ICP, GeoTransformer [20], and PREDATOR [6]. All received the same isolated XYZ observation, BIM-derived geometry, and acquisition-side information before method-specific preprocessing. Known upright and side conditions were applied during candidate verification in RANSAC, Super4PCS, and GeoTransformer’s local-to-global registration (LGR), including PREDATOR’s RANSAC pose stage. For the single-output FGR and TEASER++ estimators, these conditions admitted or rejected the returned pose rather than reoptimizing it. Centroid + ICP used the common nominal upright, side-dependent gauge. No exact camera pose or translation was supplied.

The five non-learning comparison pipelines used the same point-to-plane ICP implementation with normalized target normals, a 15 mm correspondence gate, at most 100 iterations, and relative fitness/RMSE tolerances of 10^−6^. Their geometric preprocessing used 10 mm voxels and a 20 mm normal radius with at most 30 neighbors; feature-based pipelines used a 50 mm descriptor radius with at most 100 neighbors. GeoTransformer and PREDATOR retained published 3DMatch weights, official feature preprocessing, and native pose stages without fine-tuning or an additional ICP step. PREDATOR used a compatibility implementation of the official legacy RANSAC sampling and validation settings. Randomized classical calls used seed 480; settings were fixed across observations. Table 2 summarizes the configurations, and all methods used the same evaluation metrics.

The comparison isolates geometric registration and pretrained partial-registration performance under a common single-cloud input protocol. Non-learning baselines test conventional initialization and optimization, while pretrained baselines test transfer of learned geometric correspondences without target-domain training. Complete BIM/CAD-guided welding workflows were not reproduced as additional baselines because their model alignment is coupled to different inputs or stages, such as multiview seam fusion, feature-specific segmentation, or sensor-guided trajectory correction [13,15,16]. Reproducing only their registration stage would not evaluate those complete workflows.

### 4.2. Synthetic GT Benchmark and Results

The fixed synthetic benchmark was generated from ten BIM models of strong-axis H-section beam–column joints created in Tekla Structures. The IFC models were converted into BIM-derived OBJ and USD assets using Blender/Bonsai and the Pixar USD API, imported into NVIDIA Isaac Sim, and uniformly instantiated at scale factors of 0.8, 1.0, and 1.2. This produced 30 BIM-scale assets. A total of 201 partial, joint-facing depth views were selected from frontal and oblique viewpoints on both sides of the joint, with camera heights ranging from 0.96 m below to 0.96 m above the joint center.

Each viewpoint produced matched Raw and Noise-corrupted observations with identical BIM identity, scale, camera pose, and intrinsics, resulting in 402 observations arranged as 201 pairs. The Noise-corrupted condition sequentially applied incidence-angle-dependent dropout, scattered dropout, and Gaussian depth perturbation. Their activation probabilities were 0.80, 0.88, and 0.95, respectively; the incidence-angle term used an exponent of 1.5 and a modulation factor of 1.1, the scattered-dropout rate ranged from 0.005 to 0.015, and the depth-noise standard deviation ranged from 1.8 to 3.8 mm. The nominal BIM configurations are summarized in Table 3.

The synthetic benchmark evaluated the upper- and lower-flange nominal seam paths stored in each BIM-derived asset; the vertical web-joint path was not present in this cohort. We report sample-weighted nominal-seam RMSE, an area-weighted RMS form of the ADD model-displacement measure [30], and geodesic relative rotation error (RRE) on SO(3) [25]. Let $G_{\mathrm{syn}}$ denote the two evaluated paths, $s_{g,j}$ the jth sample on path g, $M_{g}$ its sample count, M the total sample count, and $A_{B}$ the evaluated BIM surface area:

(5a) $$E_{\mathrm{seam}}=1000\sqrt{\frac{1}{M}\underset{g\in G_{\mathrm{syn}}}{\sum }\overset{M_{g}}{\underset{j=1}{\sum }}{\left\|\hat{T}(s_{g,j})-T_{\mathrm{GT}}(s_{g,j})\right\|}_{2}^{2}}\;[\mathrm{mm}],$$

(5b) $$E_{\mathrm{ADD}}=1000\sqrt{\frac{1}{A_{B}}\int_{S_{B}}{\left\|\hat{T}(p)-T_{\mathrm{GT}}(p)\right\|}_{2}^{2}dA}\;[\mathrm{mm}],$$

(5c) $$E_{\mathrm{RRE}}=\frac{180}{\pi }{cos}^{-1}[clip(\frac{tr(\hat{R}{R_{\mathrm{GT}}}^{T})-1}{2},\;-1,\;1)]\;[\deg]$$

The estimated and ground-truth transformations are denoted by $\hat{T}$ and TGT, respectively. Lower values indicate better accuracy. Error quantiles used valid pose estimates, whereas the fixed 5 and 10 mm fractions retained all 402 observations, including no-pose outcomes, in the denominator. See Equation (5a–c) and Table 4.

**Figure 3:**
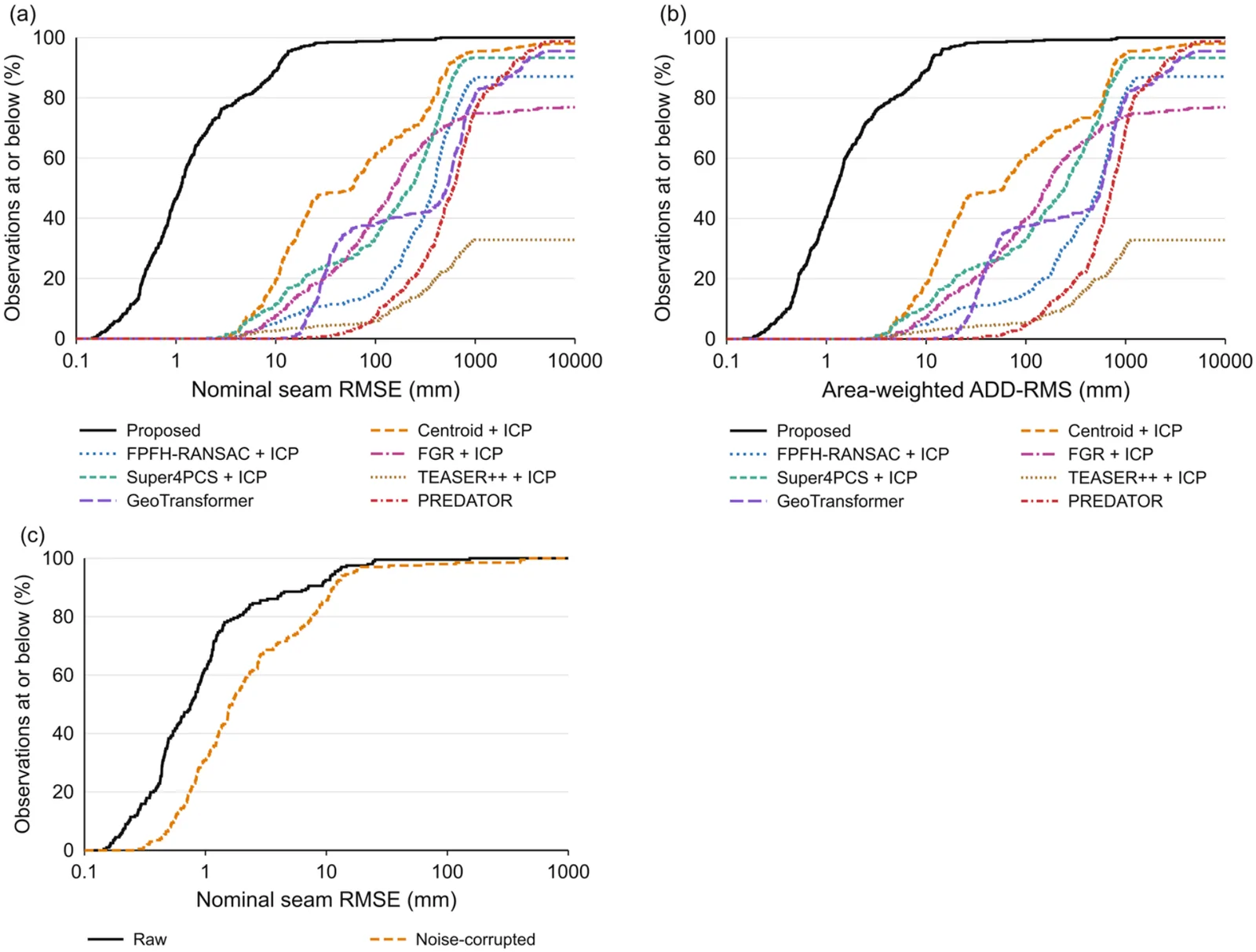
Empirical error distributions on the synthetic benchmark: (**a**) nominal-seam RMSE, (**b**) area-weighted ADD-RMS, and (**c**) proposed-method seam RMSE by paired condition. Curves retain the full denominator. The distance axes are logarithmic; errors above the displayed range remain in the denominator and are not moved to the axis boundary. No-pose outcomes contribute no finite value.

The proposed method returned a finite pose for all 402 observations and achieved a median nominal-seam RMSE of 1.110 mm, a q95 of 13.213 mm, a median ADD-RMS of 1.249 mm, and a median RRE of 0.067°. The fractions within 5 and 10 mm were 80.85% and 89.05%, respectively. Figure 3 shows that the advantage over the evaluated comparison methods extended across most of the observed error distribution rather than appearing only at the median.

The non-learning comparison methods localized 11–79 observations within 10 mm, compared with 358 for the proposed method. GeoTransformer and PREDATOR did not produce a seam estimate within 10 mm; their median seam RMSE values were 479.306 and 591.080 mm, respectively. No-pose counts were zero for centroid + ICP, 50 for FPFH–RANSAC + ICP, 75 for FGR + ICP, 15 for Super4PCS + ICP, 265 for TEASER++ + ICP, 18 for GeoTransformer, and five for PREDATOR. For FGR and TEASER++, these counts include outputs rejected by the direction conditions; a lower finite-only median must therefore not be interpreted as improved pose accuracy on all observations. Large errors, including numerical ICP failures, remain in the statistics. Five proposed estimates exceeded 100 mm, with a maximum of 451.411 mm: three had near-half-turn rotation errors and two were predominantly translational.

Table 5 separates the paired Raw and Noise-corrupted conditions. Median seam RMSE increased from 0.761 mm for Raw observations to 1.667 mm for Noise-corrupted observations. The within-5-mm fraction decreased from 88.56% to 73.13%, and the within-10-mm fraction from 92.54% to 85.57%. A two-sided Wilcoxon signed-rank test [31] comparing the proposed method’s seam RMSE across the 201 matched pairs indicated a statistically significant increase after noise addition: errors increased in 164 pairs and decreased in 37, with a median paired increase of 0.529 mm (W = 2740, *p* < 0.001). A complementary analysis using one median paired difference per BIM identity also showed positive differences for all ten identities (exact two-sided *p* = 0.00195). These results indicate that added noise reduced localization accuracy, particularly under the stricter error threshold, although most Noise-corrupted observations remained within 10 mm.

### 4.3. Laboratory Benchmark and Results

**Figure 4:**
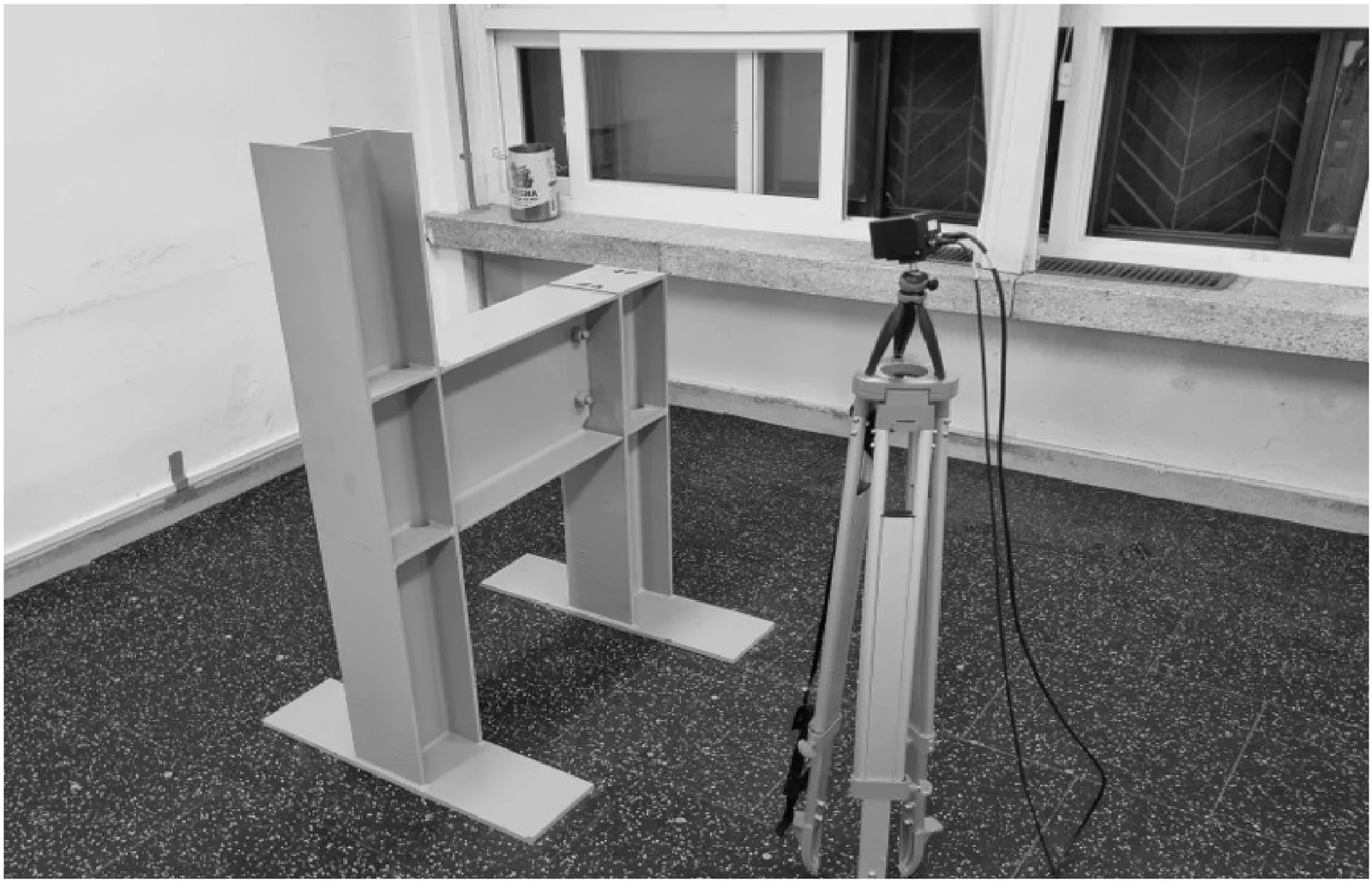
Laboratory experimental setup. The assembly consists of the target column visible on the left and the support column visible on the right.

The laboratory benchmark comprised 180 single-frame observations of one physical strong-axis H-section beam–column assembly. A Lucid Vision Labs Helios2 Ray Outdoor time-of-flight camera was aimed at the joint from two opposing sides (Side A and Side B), at nominal joint-referenced distances of 0.6 and 0.8 m, azimuths of −30°, 0°, and +30°, and heights of 0.5, 0.8, and 1.1 m. Six view groups with five repeated shots were acquired within each side–height condition, giving 2 × 3 × 6 × 5 = 180 observations. The within-group distance–azimuth acquisition order was not retained; groups are therefore treated as anonymous rather than assigned retrospective angle labels. The nominal assembly dimensions are listed in Table 6, and the laboratory setup is shown in Figure 4.

All eight methods were evaluated on the same 180 observations. To represent use without a ground-truth pose reference, laboratory performance was assessed through compatibility between the observed point cloud and transformed BIM-derived member surfaces. These measures complement the synthetic pose and nominal-seam errors.

Following the one-sided closest-surface principle used in geometric registration [17], surface compatibility was measured from each preprocessed observation point x_i_ to the transformed source BIM beam-and-column surface mesh $S_{BC}$, excluding joint-detail surfaces. This evaluation mesh retains the original member geometry rather than the analytic fitting union $S_{B}$. The evaluation distances $d_{i}$ and thresholds τ below are reported in millimeters; they are distinct from the meter-valued fitting distance $d_{i}$(T) and signed translation coordinate τ in Appendix A. For N evaluated points, the scene-level metrics were defined as follows:

(6a) $$d_{i}=1000\underset{p\in \hat{T}(S_{\mathrm{BC}})}{min}{\left\|x_{i}-p\right\|}_{2}\;[\mathrm{mm}],$$

(6b) $$M={median}_{1\leq i\leq N}(d_{i}),\;P_{95}=Q_{0.95}({\left\{d_{i}\right\}}_{i=1}^{N}),$$

(6c) $$A_{\tau }=\frac{100}{N}\sum_{i=1}^{N}1[d_{i}\leq \tau ]\;[\%],\;\tau \in \{2,\;5\}\;\mathrm{mm},$$

(6d) $${RMS}_{5}=\sqrt{\frac{\sum_{i:d_{i}\leq 5\mathrm{mm}}{d_{i}}^{2}}{\#\{i:d_{i}\leq 5\mathrm{mm}\}}}\;[\mathrm{mm}]$$

*M* and *P*_95_ summarize the central and upper-tail point-to-surface distances, *A_τ_* is the fraction within threshold *τ*, and RMS_5_ is the conditional RMS within 5 mm; RMS_5_ remains undefined if no point satisfies that threshold. Dataset values are medians of the scene-level metrics. Lower *M*, *P*_95_, and RMS_5_ and higher *A_τ_* indicate better surface compatibility, and the one-sided formulation accommodates partial views. See Equation (6a–d).

The proposed method returned a finite pose for all 180 observations and achieved a median scene-level surface distance of 2.137 mm, a median scene P95 of 19.490 mm, and a median A5 of 82.04%. Centroid + ICP also performed well under the surface-compatibility measures. Under the stricter 2 mm criterion, however, the proposed method achieved a median A2 of 47.47%, exceeding centroid + ICP by 10.01 percentage points.

**Figure 5:**
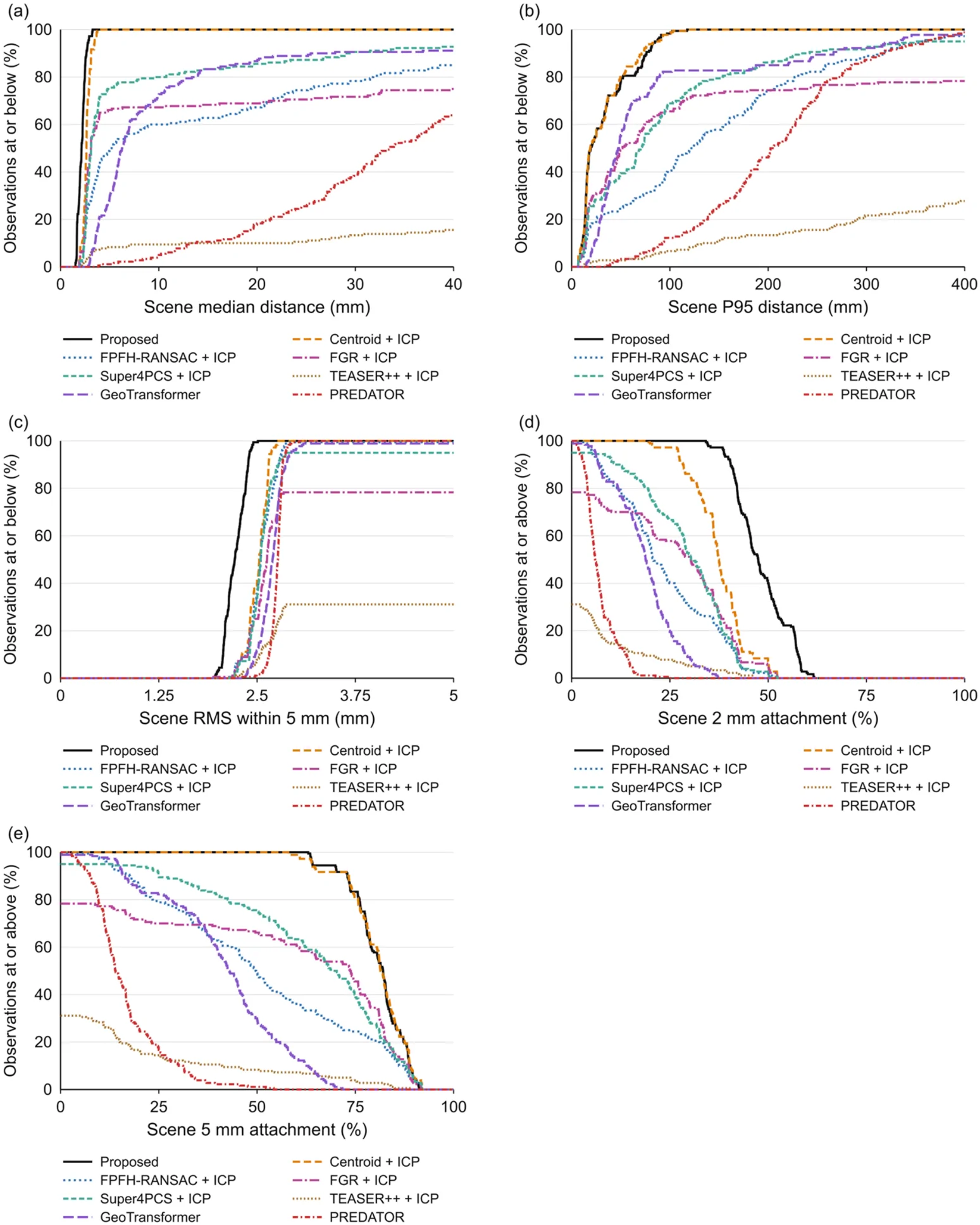
Laboratory surface-compatibility distributions for the eight final pipelines: (**a**) scene median distance, (**b**) scene P95 distance, (**c**) scene RMS within 5 mm, and (**d**,**e**) 2 and 5 mm attachment. Distance panels show the fraction at or below the horizontal value; attachment panels show the fraction at or above it. Every curve uses all 180 observations as its denominator. No-pose outcomes contribute no finite value.

Figure 5 presents the complete distribution of the five laboratory surface-compatibility metrics.

The median surface distance was 6.237 mm for GeoTransformer and 33.551 mm for PREDATOR, compared with 2.137 mm for the proposed method; their median 5 mm attachment values were 43.06% and 13.97%. No-pose counts were one for FPFH–RANSAC + ICP, 39 for FGR + ICP, nine for Super4PCS + ICP, 124 for TEASER++ + ICP, and two for GeoTransformer; the other methods returned all 180 poses. Table 7 reports finite-pose medians, while Figure 5 retains all 180 observations in each curve’s denominator.

The vertical web-joint path was additionally assessed through local surface compatibility. For each of the 180 proposed estimates, observed points within a 20 mm tube around the transformed nominal path were evaluated using the same source surface mesh and Equation (6a–d). This reporting radius is four times the 5 mm attachment distance, with 10 and 30 mm checks to examine neighborhood dependence. All observations contained points at 20 mm; the median count was approximately 4178. The median of scene-level surface medians was 2.763 mm, the median scene P95 was 5.758 mm, and the median 2 and 5 mm attachment values were 35.50% and 86.19%. The 10 and 30 mm checks gave median surface distances of 2.154 and 2.587 mm, respectively; six observations had no points in the 10 mm tube. These measurements describe observed surfaces around the estimated vertical path, conditional on its pose, rather than an independently measured weld-root error.

**Figure 6:**
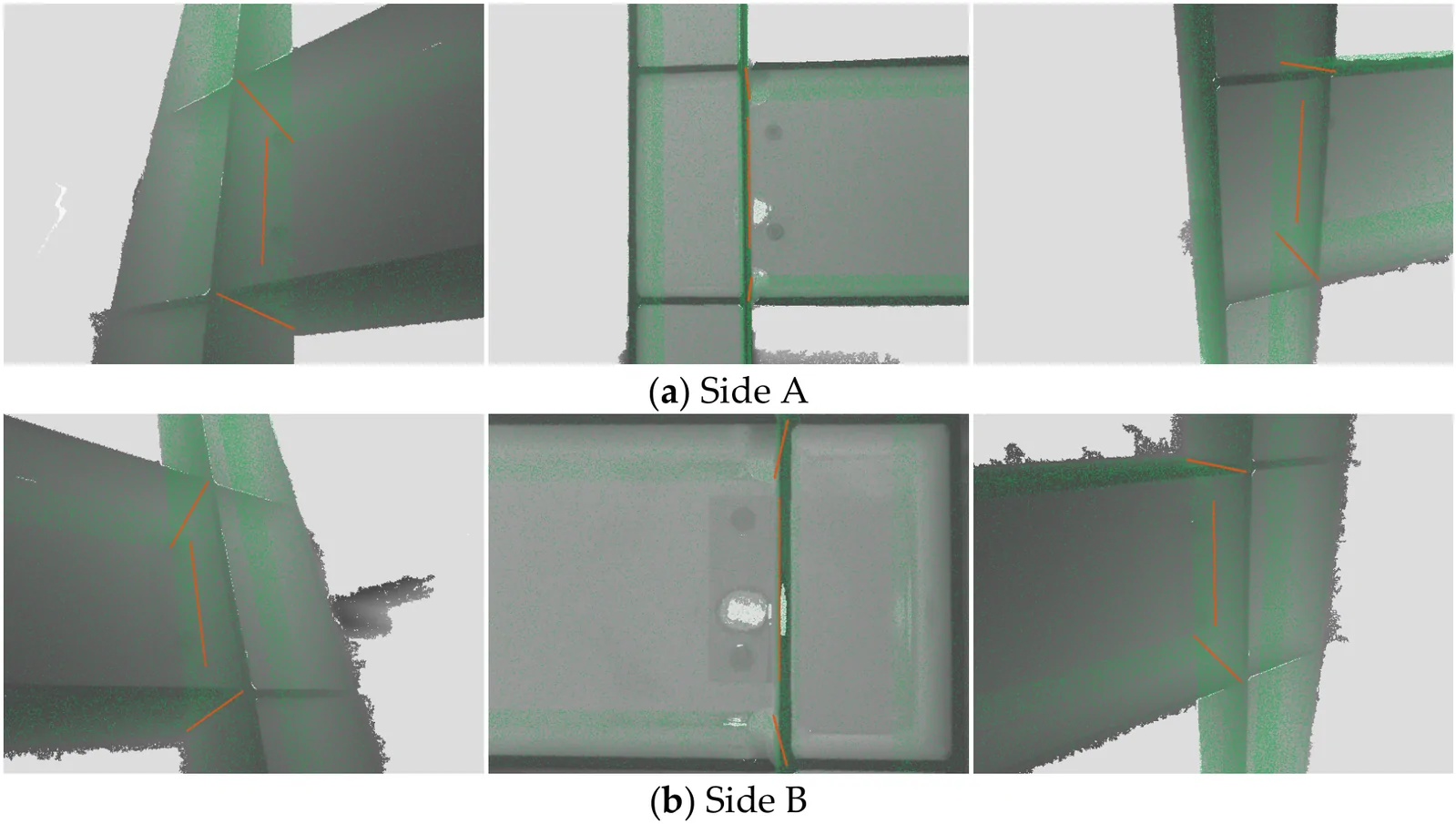
Qualitative laboratory overlays across representative partial views from both acquisition sides: (**a**) Side A at nominal camera heights of 0.5, 0.8, and 1.1 m, respectively, and (**b**) Side B at the corresponding heights. Gray, green, and orange denote the observed point cloud, transformed BIM-derived surfaces, and localized nominal seam paths, respectively.

Figure 6 shows six representative proposed-method results from both acquisition sides, including the upper, lower, and vertical nominal seam paths.

### 4.4. Ablation, Sensitivity, and Computational Analysis

Three complementary analyses were conducted. First, component ablations replaced the initialization strategy, removed finite patch boundaries, or replaced the Welsch mixture, with the other processing stages retained. Second, numerical sensitivity was assessed by perturbing the depth-cover radii and sample caps; alternative terminal risks were also compared by rescoring identical refined poses. These quality comparisons used all 402 synthetic observations. Third, computational analysis combined candidate and iteration counts from the complete synthetic and laboratory cohorts with repeated warm timing on a matched 12-observation panel. The proposed 6000-point initialization and 6000-point refinement setting was the common baseline. Table 8 and Table 9 report seam RMSE and fixed-tolerance fractions; quantiles use finite poses, whereas threshold denominators retain all observations.

#### 4.4.1. Ablation Study

To compare initialization strategies, the basic pre-ICP pose estimate from each non-learning comparison method, obtained without direction conditioning, was passed to the proposed refinement, replacing its existing ICP refinement. Thus, each conventional initial estimate and every member of the proposed initial set underwent the same finite-surface optimization. This comparison evaluates single-estimate initialization against the proposed BIM-coordinate-supported multi-pose initialization, rather than isolating candidate count. The within-10-mm fraction was 1.74–14.18% for these replacements, compared with 89.05% for the proposed method. Removing finite patch boundaries increased median seam RMSE from 1.110 to 3.817 mm and reduced the within-10-mm fraction to 69.15%, demonstrating the contribution of bounded member dimensions.

Replacing the mixture with a single 5 mm Welsch loss, while retaining all three action-limit stages, improved median RMSE to 0.881 mm but increased q95 to 25.799 mm and reduced the within-10-mm fraction to 80.35%. Terminal ordering was tested separately on identical refined poses. Ranking by the mixture instead of the 2 mm risk reduced the within-10-mm fraction from 89.05% to 68.16%. The mixture therefore provided a better balance between central precision and larger errors during optimization, whereas the narrower terminal risk more effectively selected among the resulting placements.

#### 4.4.2. Parameter Sensitivity

The cover-radius perturbations assessed whether initialization depended strongly on a particular depth subdivision. Scaling all radii by 0.75 or 1.25 gave median RMSE values of 1.077–1.127 mm and within-10-mm fractions of 88.06–89.05%, including the proposed setting. This limited variation supports local robustness of the discretization, rather than identifying a uniquely optimal radius. Reducing the coarse sample cap to 2048 or 4096 points lowered the within-10-mm fraction to 84.08% or 86.32%. Increasing the refinement sample cap to 12,000 or 24,000 points, with initialization fixed, reduced median RMSE to 1.023 or 1.047 mm. The 24,000-point setting increased the within-2-mm fraction from 68.41% to 70.65% but did not improve q95 or the within-5-mm fraction.

Terminal-bandwidth sensitivity was evaluated without rerunning refinement. A 1 mm risk improved median precision but increased q95 to 17.802 mm; a 5 mm risk reduced the within-10-mm fraction to 77.86%. Together with the sampling results, these comparisons support 6000/6000 points and a 2 mm terminal bandwidth as a balanced configuration for the evaluated task, rather than a universally optimal parameter choice.

#### 4.4.3. Computational Work and Processing Time

Computational analysis examined how the number of proposed placements and the refinement workload translated into end-to-end processing time. Initialization examines the 24 proper signed permutations once; the median number of emitted rotations was 12 in both cohorts. With K finite rectangles, $N_{R}$ refinement points, $M_{0}$ initial poses, and I iterations per candidate, direct finite-surface evaluation scales as O(K$N_{R}M_{0}$I), apart from line-search evaluations and fixed-size pose solves. Table 10 reports actual candidate counts and iteration counts over the complete cohorts, together with timing on a matched panel. The median initial-set sizes were 552 and 586, and the median per-candidate iteration totals were 43 and 91 for synthetic and laboratory inputs, respectively. Early stopping reduced these totals below the 192-iteration maximum.

Warm processing time was measured on six synthetic and six laboratory observations using 24 warm-up calls followed by 48 measured calls. Timing covered sampling, initialization, refinement, selection, and seam transfer from prepared XYZ and BIM assets, excluding acquisition, external preprocessing, disk access, offline BIM preparation, first compilation, evaluation, and rendering. Median total times were 2.517 s and 3.180 s, respectively, corresponding to reciprocal rates of 0.397 and 0.314 observations/s (FPS and Hz). Laboratory preparation and refinement medians were 0.516 s and 2.577 s; refinement was therefore the principal measured computational cost. Laboratory total q95 and maximum times were 3.667 s and 3.746 s. These results characterize the matched timing panel, not the latency distribution of the entire benchmark.

## 5. Discussion

### 5.1. Roles of Initialization, Refinement, and Terminal Comparison

The combined results indicate that accurate nominal-seam transfer requires initialization that accommodates repeated member surfaces as well as local surface fitting. Applying the proposed refinement to conventional initial estimates did not reproduce the accuracy obtained from its BIM-coordinate-supported pose set. This difference concerns the complete initialization strategy: alternative placements are retained until each has been locally optimized. The infinite-plane comparison further demonstrates that flange and web boundaries help constrain placements that would otherwise agree with unbounded supporting planes. The conventional and pretrained comparisons provide complementary evidence. Under the tested input conditions, neither generic geometric estimators nor transferred GeoTransformer and PREDATOR models consistently resolved the repeated surfaces of the connected assembly. Known side and upright conditions restrict orientation but do not determine the correct translation. The pretrained results specifically concern published 3DMatch models used without fine-tuning, rather than learned registration methods in general.

The ablation results also distinguish the role of the fitting objective from that of terminal ordering. The mixture retains useful responses over several residual ranges during optimization; its benefit is reflected in fewer large localization errors than with the single 5 mm loss, despite a less favorable median. Once optimization is complete, the narrower terminal risk discriminates close surface agreement more effectively: applying mixture-based ordering to the same endpoints loses 84 within-10-mm estimates. This separation allows the optimization objective to accommodate initial offsets without imposing the same tolerance on final pose comparison. Because both representations and updates are computed directly from BIM geometry, changes to nominal dimensions or seam paths do not require training annotations or network retraining within the supported model class.

### 5.2. Failure Modes and Validation Scope

The five synthetic errors exceeding 100 mm comprised three near-half-turn orientation errors and two predominantly translational misalignments. These outcomes demonstrate that local agreement with repeated surfaces can coexist with substantial displacement of the nominal seam paths. A low residual alone is therefore insufficient to resolve every admissible placement. Additional observations that expose member boundaries or connection details, or an independent positional measurement, offers a principled means of verification when such ambiguity matters to the subsequent task. Evaluating these verification strategies is preferable to treating a small score gap as evidence that a pose is physically correct.

The synthetic benchmark covers ten BIM identities and three scales, whereas the laboratory observations comprise repeated partial views of one physical assembly. The latter demonstrate transfer to measured geometry but do not quantify variability across independently fabricated specimens. Similarly, the synthetic seam error directly evaluates the two flange paths, while the vertical-path analysis measures local surface compatibility around the estimated path. Additional specimens and independently measured seam references are needed to assess fabrication variability and all active seam classes on a common accuracy basis.

### 5.3. Practical Application and BIM-to-Built Differences

The method is intended to establish seam locations before a welding operation, when an incorrect localization can have consequences that outweigh a short computational delay. The measured processing interval of approximately 2.5–3.2 s is consistent with this task-level role, rather than continuous torch tracking, although its suitability must be assessed within the complete sensing and welding cycle. Further reduction of multi-pose refinement cost would allow more frequent verification updates. The demonstrated performance also depends on target isolation, calibration, and the stated acquisition conditions; these remain system-level requirements rather than capabilities established by the registration experiments.

BIM-to-built differences affect both the surface fit and the interpretation of the transferred paths. Rigid assembly displacement is represented by the estimated pose, whereas dimensional changes, local deformation, and altered connection details remain model discrepancies. Fabrication and erection controls motivate the use of nominal member faces, but they do not establish a seam-localization tolerance. Robust fitting limits the influence of inconsistent samples without determining whether the supplied BIM adequately represents the workpiece. The transferred paths can therefore support subsequent inspection or finer sensing, while verification against the actual joint remains necessary where model discrepancies could affect the welding operation.

## 6. Conclusions

This study presented a non-learning BIM-guided method for localizing nominal weld seams from a partial point cloud of an H-section beam–column joint. Rather than requiring the seam to be directly observed, the method estimates the joint pose from the visible flange and web geometry. An observation-conditioned set of initial poses is generated from projective measurements and BIM-derived geometric representations, after which every pose is refined using the same robust finite-surface objective. The selected final pose is then used to transfer all active BIM-defined nominal seam paths into the observation coordinate system without learned seam labels or missing-shape reconstruction.

In the synthetic benchmark, the proposed method returned a pose estimate for every observation and achieved median nominal-seam RMSE, ADD-RMS, and RRE values of 1.110 mm, 1.249 mm, and 0.067°, respectively, with 89.05% of seam estimates within 10 mm. In the laboratory benchmark, it returned a pose for every observation and achieved a median point-to-surface distance of 2.137 mm and a median 5 mm attachment of 82.04%. The stricter attachment results also indicated denser local surface agreement than that of the evaluated comparison methods. Warm processing medians on the matched timing panel were 2.52 s and 3.18 s for synthetic and laboratory inputs, respectively. These results support the proposed approach to localizing nominal BIM weld seams from partial observations.

The study establishes a geometric route from partial member observations to nominal BIM seam locations without supervised seam detection or reconstruction of missing surfaces. Further research should reduce dependence on prescribed acquisition conditions and resolve repeated-surface ambiguity through richer observations and explicit verification. Broader validation across connection geometries and independently measured as-built variation, together with more efficient computation, will be needed to extend this approach to less controlled welding environments.

## Figures and Tables

**Table 1:** Comparison of sensing, seam visibility, model and training requirements, outputs, and validation in the reviewed studies.

Strategy/Studies	Sensing/Views	Seam Visibility	3D Reference	Learning	Training GT	Output	Validation
Local seam geometry [3,4,11,12]	Stereo RGB [11]; local/partial XYZ [3,4,12]	Visible seam edges or adjoining faces	Not used	No	None	Observed seams	Real: plate/groove workpieces and RGB-D scans [3]. Synthetic: washers [3].
Learned seam detection [5,14]	RGB-D, single view [5]; RGB, stitched views [14]	Visible edges; guide symbols [14]	Not used	Yes	Keypoints [5]; NR [14]	Observed seams	Real: straight/curved-seam parts [5]; steel weldments [14].
Model/multiview guidance [13,15,16]	RGB-D partial views merged [13]; XYZ [15,16]	Visible edges [13,15]; laser profile for correction [16]	None [13]; model option [15]; BIM [16]	No	None	Observed [13,15]; nominal [16]; pose [15,16]	Real: multiseam parts [13]; pipe/plate/groove joints [15]; intersecting joints [16].
Geometric registration [7,8,17,18,19]	XYZ pair; partial overlap	N/A: no seam output	Point set (CAD optional)	No	None	Pose [7,8,17,19]; FPFH descriptors only [18]	Synthetic/real: general objects and scene scans, including public datasets.
Learned registration [6,20]	XYZ pair; low overlap	N/A: no seam output	Point set (CAD optional)	Yes	Known training poses	Pose	Synthetic: ModelNet objects. Real: indoor/outdoor scans (public datasets).
Proposed	XYZ; single partial view	Seam may be unobserved	BIM + nominal seam paths	No	None	Pose; nominal seams	Synthetic: 30 BIM assets. Real: 180 scans of one steel beam–column assembly.

XYZ includes RGB-D-derived point clouds. Training GT denotes supervision, not a test-time pose or seam input; “None” means no supervised training stage, not no evaluation reference. A CAD/BIM model is prior geometry, not as-built GT. NR: training-label details not established from the available description. Visibility entries identify the edges or joint faces used for extraction; combining views is distinct from estimating an unobserved path from one view.

**Table 2:** Fixed comparison-method settings. Geometric preprocessing and direction conditioning are described in Section 4.1. Learned methods retain their native pose stages without added ICP.

Component	Configuration
FPFH–RANSAC	Mutual FPFH; 15 mm gate; 100,000 iterations; confidence 0.999; upright/side candidate support
FGR	5 mm correspondence distance; fixed optimizer settings; upright/side admission of its single output
Super4PCS	Overlap 0.2; delta 15 mm; 200 samples; search budget 10 s; upright/side verification
TEASER++	Mutual FPFH; 15 mm noise bound; fixed scale; GNC-TLS rotation, at most 100 iterations; upright/side single-output admission
GeoTransformer	Published 3DMatch weights; official multiscale feature preprocessing; native LGR with upright/side candidate support; no added ICP
PREDATOR	Published 3DMatch weights; official neighbor caps 38/35/36/39; learned matching then RANSAC with upright/side support; 50,000 iterations and 1000 validations; no added ICP
Canonical added ICP	Non-learning comparison methods only; normalized target normals; point-to-plane; 15 mm; at most 100 iterations; relative fitness/RMSE 10^−6^

**Table 3:** Nominal BIM configurations used in the synthetic benchmark.

BIM Identity	Column Section ($H\times B\times t_{w}\times t_{f}$) (mm)	Beam Section ($H\times B\times t_{w}\times t_{f}$) (mm)	Web Plate (t × h×b) (mm)
SWB_001	175×175×7.5×11	350×175×7.5×11	11×225×130
SWB_002	200×200×8×12	400×200×8×13	11×290×90
SWB_003	200×200×8×12	450×200×9×14	12×350×90
SWB_004	300×300×10×15	500×200×10×16	12×365×130
SWB_005	300×305×15×15	600×200×11×17	12×465×130
SWB_006	350×350×12×19	612×202×13×23	15×480×120
SWB_007	300×300×10×15	588×300×12×20	14×420×120
SWB_008	300×300×10×15	700×300×13×24	14×530×120
SWB_009	400×400×13×21	800×300×14×26	15×620×120
SWB_010	400×400×13×21	900×300×16×28	17×710×130

**Table 4:** Overall synthetic benchmark comparison.

Method	Finite Poses	Seam q50 (mm)	Seam q95 (mm)	ADD-RMS q50 (mm)	RRE q50 (°)	Seam ≤ 5 mm (%)	Seam ≤ 10 mm (%)
Proposed	402/402	1.110	13.213	1.249	0.067	80.85	89.05
Centroid + ICP	402/402	58.540	826.997	59.392	0.880	5.97	19.65
FPFH–RANSAC + ICP	352/402	327.999	805.915	453.023	35.260	1.49	5.22
FGR + ICP	327/402	97.949	$3.547\times 10^{9}$	107.608	0.952	1.74	7.71
Super4PCS + ICP	387/402	200.173	736.463	222.581	0.948	5.22	11.44
TEASER++ + ICP	137/402	341.834	908.693	422.479	37.398	1.49	2.74
GeoTransformer	384/402	479.306	2958.046	547.587	3.925	0.00	0.00
PREDATOR	397/402	591.080	3113.803	722.873	40.795	0.00	0.00

Table 4 reports each fixed final pipeline, not the best result chosen per observation. Finite-pose counts exclude no-pose outcomes; threshold fractions use all 402 observations.

**Table 5:** Proposed-method results for the paired synthetic input conditions.

Condition	n	Seam q50 (mm)	Seam q95 (mm)	ADD-RMS q50 (mm)	RRE q50 (°)	Seam ≤ 5 mm (%)	Seam ≤ 10 mm (%)
Raw	201	0.761	11.780	0.858	0.047	88.56	92.54
Noise-corrupted	201	1.667	15.751	1.960	0.111	73.13	85.57

**Table 6:** Nominal dimensions of the laboratory assembly.

Component	Nominal Dimensions (mm)	Length (mm)	Material	Quantity
Target column	H—200×200×8×12	1500	SS275	1
Beam	H—400×200×8×13	600		
Web plate	250×80×10	-		

**Table 7:** Overall laboratory surface-compatibility results.

Method	Finite Poses	M (mm)	P95 (mm)	RMS5 (mm)	A2 (%)	A5 (%)
Proposed	180/180	2.137	19.490	2.216	47.47	82.04
Centroid + ICP	180/180	2.632	18.919	2.527	37.46	81.46
FPFH–RANSAC + ICP	179/180	5.082	120.167	2.547	20.84	49.76
FGR + ICP	141/180	2.761	36.934	2.564	34.00	77.57
Super4PCS + ICP	171/180	3.090	65.633	2.538	31.85	70.99
TEASER++ + ICP	56/180	41.002	245.879	2.671	8.85	20.51
GeoTransformer	178/180	6.237	47.171	2.695	18.97	43.06
PREDATOR	180/180	33.551	204.031	2.764	5.97	13.97

Table 7 reports medians over finite poses. All 180 observations, including no-pose cases, remain in the Figure 5 denominator. RMS5 was defined for every returned pose in the final eight pipelines; no-pose outcomes were not assigned a zero residual.

**Table 8:** Structural comparisons on all 402 synthetic observations. Errors are nominal seam RMSE.

Configuration	n	q50 (mm)	q95 (mm)	Max (mm)	≤2 mm	≤5 mm	≤10 mm
Proposed	402	1.110	13.213	451.411	68.41%	80.85%	89.05%
Centroid + proposed refinement	402	25.511	621.665	904.939	0.00%	0.75%	12.19%
FPFH–RANSAC + proposed refinement	402	448.202	864.345	1160.929	1.00%	1.49%	2.99%
FGR + proposed refinement	402	99.840	637.395	1077.152	0.25%	1.99%	14.18%
Super4PCS + proposed refinement	386	433.143	955.744	1317.424	0.25%	0.75%	5.22%
TEASER++ + proposed refinement	402	507.377	962.670	1280.225	0.00%	0.50%	1.74%
Infinite supporting planes	402	3.817	203.666	627.042	41.79%	55.97%	69.15%
Single J5 refinement loss	402	0.881	25.799	424.898	65.17%	72.39%	80.35%
Terminal Jmix	402	2.808	23.019	967.346	45.52%	53.98%	68.16%

All threshold fractions retain all 402 observations; n denotes finite poses. The proposed row is repeated for comparison.

**Table 9:** Parameter sensitivity on all 402 synthetic observations. Errors are nominal seam RMSE.

Configuration	n	q50 (mm)	q95 (mm)	Max (mm)	≤2 mm	≤5 mm	≤10 mm
Proposed	402	1.110	13.213	451.411	68.41%	80.85%	89.05%
Cover radii ×0.75	402	1.077	16.334	475.802	69.15%	79.60%	88.31%
Cover radii ×1.25	402	1.127	15.415	475.802	66.67%	77.36%	88.06%
Coarse 2048/refine 6000	402	1.246	20.829	475.801	62.44%	73.38%	84.08%
Coarse 4096/refine 6000	402	1.215	15.703	404.267	65.17%	77.11%	86.32%
Coarse 6000/refine 12,000	402	1.023	16.203	475.476	70.15%	80.85%	88.56%
Coarse 6000/refine 24,000	402	1.047	14.138	475.802	70.65%	80.35%	89.55%
Terminal J1	402	1.061	17.802	404.267	69.90%	80.60%	88.56%
Terminal J5	402	1.587	18.083	967.346	53.73%	63.93%	77.86%

All threshold fractions retain all 402 observations; n denotes finite poses. The proposed row is repeated for comparison.

**Table 10:** Work counts on complete cohorts and warm processing time on the matched timing panel.

Measure	Synthetic	Laboratory
Complete-cohort observations	402	180
Initial poses min/median/max	317/552/2613	420/586/726
Initial poses mean	957.0	580.7
Emitted rotations min/median/max	8/12/12	12/12/12
Candidate iterations min/median/max	6/43/192	6/91/192
Matched panel observations	6	6
Median total processing (s)	2.517	3.180
Reciprocal median (FPS and Hz)	0.397	0.314
Laboratory median preparation (s)	—	0.516
Laboratory median refinement (s)	—	2.577

## Data Availability

The raw data supporting the conclusions of this article will be made available by the authors on request.

## Appendix A. Core Definitions for Observation-Conditioned Initialization

Table A1 collects the notation used in Appendix A and Appendix B, including quantities inherited from Section 3. Coordinates and distances are evaluated internally in meters, and angles in radians; reported evaluation distances and rotation errors use millimeters and degrees. In particular, the bandwidths in Equation (4b) equal those in Appendix B.2 after unit conversion, and the 2 mm terminal risk uses 0.002 m. Counts, indices, unit directions, normalized masses, and objective values are dimensionless. A rigid pose combines a dimensionless rotation with a meter-valued translation. Reused letters are distinguished by their subscripts and stated local context.

**Table A1:** Symbols, definitions, and units used in Appendix A and Appendix B.

Symbol	Definition	Unit
X $,\;X_{C}$ $,\;X_{R}$ $;\;N_{C}$ $,\;N_{R}$	Input observation and its coarse/refinement subsets; their sample counts.	m (points); count
$x_{i}$ $,\;x_{j}$ $,\;{\bar{x}}_{i}$ $;\;N_{i}$ , k	Observed points and neighborhood centroid; neighbor set and its size (at most 12).	m; set/count
$\Sigma_{i}$ $;\;\lambda_{i,0}$ $,\;\lambda_{i,1}$ $,\;\lambda_{i,2}$	Local covariance and its ordered eigenvalues; distinct from joint depth and the Welsch bandwidth set.	m^2^
$n_{i}$ $;\;r_{i}$	Sign-free unit local normal; outer-neighbor distance.	1; m
$\eta_{i}$ $;\;a_{i}$	Local area proxy; corresponding normalized area mass.	m^2^; 1
ε $;\;c_{i}$	Machine precision; directional confidence, clipped to [0, 1].	1
$\mu_{i}$ $;\;m_{i}^{unres}$	Supported directional mass and unresolved remainder of normalized area mass.	1
$\sigma_{n,i}$ $;\;\sigma_{h,i}$	Normal-angle uncertainty; signed-coordinate half-width.	rad; m
$M_{2}$ $;\;Q_{O}$ $;\;Q_{O}$	Second projective moment; representative orientation; its complete proper signed orbit.	1
P $;\;P_{3}^{+}$	Proper signed permutation matrix; the set of its 24 possible values.	1
$\Delta_{m}$ ; p	BIM-derived triangle and a point on it; m is a triangle index here.	m (vertices)
$a_{l}^{B}$ $;\;H_{ml}^{\Delta }$	Unit assembly axis l (l = 1,2,3); the triangle projection interval along it.	1; m
$R_{r}$ ; r	Retained BIM-to-observation rotation; its index.	1; index
$I_{rl}$ $;\;U_{irl}$	Directionally compatible observation-index set; merged translation-interval union for one element.	index set; m
τ $;\;D_{rl}$ $;\;\Theta_{rl}$	Signed translation coordinate, its admissible interval, and its supported subset.	m
$S_{rl}$ $;\;W_{rl}$ $;\;C_{rl}^{unres}$ $;\;R_{rl}$	Translation-support mass, available directional mass, unresolved capacity, and required mass; the last is not a rotation matrix.	1
$\delta_{rl}^{mass}$; 1[.]	Forward-summation tolerance in normalized-mass units; indicator function.	1
$b_{rl}$ $;\;e_{z}$ $;\;\alpha_{rl}$	Transformed assembly axis; camera optical unit axis; depth-to-coordinate slope.	1
$c_{J}$ $;\;\beta_{rl}$	BIM joint reference; intercept of the affine depth-to-coordinate map.	m
λ $;\;\Lambda_{rl}$ $;\;\Lambda_{r}$	Positive joint depth; axiswise support set; intersection over the three axes.	m
a , b ; ρ	Endpoints of a connected depth interval; midpoint-cover radius.	m
$n_{\rho }$; j; $P_{\rho }$; p (phase)	Subdivision count and index; normalized midpoint-phase set; one phase. Here p is a scalar, not a BIM point.	count/index; 1
T , U ; R , t	Rigid poses; their rotation and translation components.	R: 1; t: m
$T_{0}^{m}$ $,\;T_{F}^{m}$ $;\;M_{0}$	Initial and terminal poses for candidate m (shown as a parenthesized superscript in the equations); number of emitted candidates.	pose; count
q ; k , K	BIM-frame query; finite-rectangle index and number of rectangles.	m; index/count
$o_{k}$ $;\;n_{k}$ $,\;u_{k}$ $,\;v_{k}$	Rectangle reference point; orthonormal normal, tangent, and longitudinal directions.	m; 1
α , β (pat $\mathrm{ch});\;\alpha_{k,min}$ $,\;\alpha_{k,max}$ $;\;\beta_{k,min}$ $,\;\beta_{k,max}$	Relative in-plane rectangle parameters and their lower/upper bounds from Equation (2b); not the depth-map coefficients.	m
$d_{k}$ $;\;h_{k}$ $,\;s_{k}$ $,\;l_{k}$	Rectangle plane level; normal, tangent, and longitudinal query projections.	m
${\bar{s}}_{k}$ $,\;{\bar{l}}_{k}$ $;\;p_{k}$ $;\;\delta_{k}^{2}$	Clamped absolute projected coordinates; closest rectangle point; squared distance to rectangle k.	m; m; m^2^
$S_{B}$$;\;d_{i}$(T)	Union of finite BIM patches; distance from inverse-transformed observation i to that union.	m (points); m
Σ; σ; |Σ|	Welsch bandwidth set; one bandwidth; the set cardinality (three).	m; m; count
$w_{i}$ (T $);\;k_{i}$	Derivative-induced IRLS factor; selected closest-rectangle index.	m^−2^; index
$\chi_{u,i}$$,\;\chi_{v,i}$$;\;P_{i}$(T)	Binary finite-bound clamp indicators; selected-feature projector.	1
$A_{i}$ $;\;j_{i}$	Query Jacobian and scalar-distance gradient in the meter-valued local action chart.	1
g(T); H(T)	Objective gradient and local Gauss–Newton/IRLS matrix in that same chart.	m^−1^; m^−2^
$V_{B}$; v$;\;|V_{B}$$|;\;d_{B}$(T,U)	Unique corner set, one corner, its cardinality, and RMS displacement between poses.	m (corners); count; m
$J_{mix}$(T$);\;J_{2}$(T)	Fixed Welsch-mixture objective; terminal Welsch risk with 0.002 m bandwidth.	1
$\hat{m}$ $;\;\hat{T}$ $;\;\hat{R}$ $,\;\hat{t}$	Smallest minimizing candidate index; selected pose and its rotation/translation.	index; pose
$A_{B}$; g (cl*a*ss); $M_{g}$; j (s*a*m*p*le)	Active seam-class set; one class; its ordered sample count and sample index.	set/index/count
$s_{g,j}$ $;\;{\hat{s}}_{g,j}$	BIM-frame nominal seam sample; its localized observation-frame sample.	m
$A_{B}$	Total BIM-surface area in Equation (5b), distinct from the active seam-class set.	m^2^

### Appendix A.1. Local Projective Measurements

A deterministic priority sample drawn once without replacement gives XC ⊆ XR ⊆ X. The projective-frame estimate and refinement use equal point mass; translation support uses the normalized local-area masses defined below. For coarse point $x_{i}$, $N_{i}$ is its k-nearest-neighbor set within XC, *k* = min(12, NC), and ${\bar{x}}_{i}$ is the neighborhood centroid. The local covariance is

(A1) $$\Sigma_{i}=\frac{1}{k}\sum_{x_{j}\in N_{i}}\left(x_{j}-{\bar{x}}_{i}\right){\left(x_{j}-{\bar{x}}_{i}\right)}^{T}$$

Let 0 ≤ *λ_i_*_,0_ ≤ *λ_i_*_,1_ ≤ *λ_i_*_,2_ be its eigenvalues. The eigenvector of *λ_i_*_,0_ gives the sign-free local normal *n_i_*. If *r_i_* is the outer-neighbor distance, the local area proxy and normalized mass are

(A2) $$\eta_{i}=\frac{\pi r_{i}^{2}}{k-1},\;\;a_{i}=\frac{\eta_{i}}{\sum_{j}\eta_{j}}$$

Let *ε* denote machine precision. The dimensionless directional confidence is

(A3) $$c_{i}=clip\left(\frac{\lambda_{i,1}-\lambda_{i,0}}{max\left(\lambda_{i,2},\;\varepsilon r_{i}^{2}\right)},\;0,\;1\right),$$

The supported and unresolved masses are then

(A4) $$\mu_{i}=a_{i}c_{i},\;\;m_{i}^{unres}=a_{i}-\mu_{i}$$

The same eigenspectrum and neighborhood radius also determine the normal-angle uncertainty and the half-width of the uncertain Hesse coordinate:

(A5) $$\sigma_{n,i}=\mathrm{atan}2(\sqrt{\lambda_{i,0}},\sqrt{\max(\lambda_{i,1},\varepsilon r_{i}^{2})})$$

(A6) $$\sigma_{h,i}=\sqrt{\lambda_{i,0}}+r_{i}\sin(\sigma_{n,i})+128\varepsilon \max(1,{∥x_{i}∥}_{2})$$

Here *ε* is dimensionless machine precision. All coordinates in Equation (A6) are evaluated numerically in meters, so the numerical floor 1 in max(1, $∥x_{i}∥$_2_) represents a 1 m reference length. Both *σ_h_*_,*i*_ and its three additive terms therefore have units of meters; *σ_n_*_,*i*_ is an angle in radians. The first two terms propagate local point and normal uncertainty, and the last accounts for floating-point coordinate scale. The angular interval controls directional compatibility, and the half-width expands the signed-coordinate interval; neither is an additional pose score.

### Appendix A.2. Projective Rotation States and BIM Hesse Atlas

The second projective moment is

(A7) $$M_{2}=\frac{1}{N_{C}}\sum_{i=1}^{N_{C}}n_{i}n_{i}^{T}$$

Its eigenvectors initialize the local SO(3) minimization in Equation (3a). If Q_O_ denotes the resulting representative and P_3_^+^ is the set of 24 signed permutation matrices with determinant +1, the complete projective orbit is

(A8) $$\left[Q_{O}\right]=\{Q_{O}P|P\in P_{3}^{+}\},$$

For each orbit state, the observation optical direction is expressed in the BIM frame. The state is retained only when its transverse-axis component agrees with the supplied acquisition-side sign. Gravity then fixes the zero-roll upright frame while preserving that viewing direction; states within the declared gravity singularity tolerance are excluded.

Let *Δ_m_* be a nondegenerate BIM-derived triangle assigned to assembly axis a_l_^B^. Its Hesse-coordinate contribution is

(A9) $$H_{ml}^{\Delta }=\left[\underset{p\in \Delta_{m}}{min}{\left(a_{l}^{B}\right)}^{T}p,\;\underset{p\in \Delta_{m}}{max}{\left(a_{l}^{B}\right)}^{T}p\right]$$

Overlapping intervals are merged independently for the three assembly axes. This atlas is compiled without observation access and is used only during initialization.

### Appendix A.3. Translation Support and Pose Materialization

For retained rotation *R_r_* and assembly-axis index ℓ ∈ {1, 2, 3}, let $I_{rl}$ contain the observation elements directionally compatible with that axis after accounting for their angular uncertainty. Each element defines an uncertain signed-coordinate interval. Its Minkowski difference with each BIM atlas interval gives a translation interval; overlapping alternatives for the same element are merged into $U_{irl}$. With τ denoting translation along the transformed axis and 1 denoting the interval indicator, the supported mass is

(A10) $$S_{rl}\left(\tau \right)=\sum_{i\in I_{rl}}\mu_{i}1\left[\tau \in U_{irl}\right],$$

The available directional mass and unresolved capacity are

(A11) $$W_{rl}=\sum_{i\in I_{rl}}\mu_{i},\;\;C_{rl}^{unres}=\sum_{i\in I_{rl}}m_{i}^{unres}$$

The mass required for compatibility is

(A12) $$R_{rl}=max\left(0,W_{rl}-C_{rl}^{unres}\right),$$

Accordingly, the supported coordinate set is

(A13) $$\Theta_{rl}=\{\tau \in D_{rl}|S_{rl}\left(\tau \right)\geq R_{rl}-\delta_{rl}^{mass}\}$$

Here *D_rℓ_* is the admissible signed-translation interval and *δ_rℓ_* mass is the dimensionless forward-summation tolerance. The support, unresolved capacity, required mass, and tolerance all use the same normalized-area convention. *R_rℓ_* is a scalar mass requirement, distinct from rotation matrix *R_r_*. Large unresolved capacity widens the admissible set rather than rejecting the rotation.

For each retained rotation, substitution of the joint-depth parameterization in Equation (3b) gives an affine signed coordinate along every transformed BIM assembly axis. The transformed axis and its two coefficients are

(A14) $$b_{rl}=R_{r}a_{l}^{B},\alpha_{rl}={b_{rl}}^{T}e_{z},\beta_{rl}=-{b_{rl}}^{T}R_{r}c_{J}$$

The corresponding positive-depth support is

(A15) $$\Lambda_{rl}=\{\lambda >0|\alpha_{rl}\lambda +\beta_{rl}\in \Theta_{rl}\},$$

The common positive-depth set is

(A16) $$\Lambda_{r}=\Lambda_{r1}\cap \Lambda_{r2}\cap \Lambda_{r3}$$

For each connected interval [*a*, *b*] and each midpoint-cover radius *ρ* ∈ {0.060, 0.045, 0.030} m, the number and normalized phases are

(A17) $$n_{\rho }=max\left(1,ceil\left(\frac{b-a}{2\rho }\right)\right)$$

(A18) $$P_{\rho }\left(\left[a,b\right]\right)=\left\{\frac{2j+1}{2n_{\rho }}:j=0,\ldots ,n_{\rho }-1\right\}$$

Duplicate phases across the three covers are removed, and *λ* = *a* + *p*(*b* − *a*) is materialized for every remaining phase *p*. All generated poses are appended in deterministic order and enter the same refinement law.

## Appendix B. Core Definitions for Finite-Surface Refinement and Selection

### Appendix B.1. Exact Projection onto a Finite Rectangle

For a BIM-frame query q and rectangle k, $o_{k}$ is a reference point and $n_{k}$, $u_{k}$, $v_{k}$ are its orthonormal normal, tangent, and longitudinal directions. The index *k* here labels a rectangle, whereas k in Appendix A.1 is the neighborhood size. The plane level and query coordinates are

(A19) $$d_{k}={n_{k}}^{T}o_{k},h_{k}={n_{k}}^{T}q,s_{k}={u_{k}}^{T}q,l_{k}={v_{k}}^{T}q.$$

The barred coordinates are obtained by clamping *s* and *l* to the rectangle’s absolute projected bounds along $u_{k}$ and $v_{k}$, respectively. These bounds include the projections of $o_{k}$ and are not merely the relative parameters in Equation (2b). With *d_k_* = $n_{k}$*^T^*$o_{k}$, all four coordinates have units of meters. The closest point is

(A20) $$p_{k}=d_{k}n_{k}+{\bar{s}}_{k}u_{k}+{\bar{l}}_{k}v_{k},$$

with squared distance

(A21) $$\delta_{k}^{2}={\left(h_{k}-d_{k}\right)}^{2}+{\left(s_{k}-{\bar{s}}_{k}\right)}^{2}+{\left(l_{k}-{\bar{l}}_{k}\right)}^{2}$$

The active finite feature is the deterministic minimum over all rectangles. Its clamp state identifies a face interior, edge, or corner.

### Appendix B.2. Welsch IRLS and Finite-Feature Model

Let Σ = {0.015, 0.0075, 0.005} m denote the bandwidth set in Equation (4b), distinct from covariance matrix Σ*i*. Its cardinality is |Σ| = 3. Writing the derivative of the per-point Welsch mixture with respect to distance as *w_i_*(*T*)$d_{i}$(*T*) gives the scalar IRLS factor

(A22) $$w_{i}\left(T\right)=\frac{1}{\left|\Sigma \right|}\sum_{\sigma \in \Sigma }\frac{exp\left[-d_{i}{\left(T\right)}^{2}/\left(2\sigma^{2}\right)\right]}{\sigma^{2}}$$

The factor *w_i_*(*T*) has units m^−2^ and is not a normalized point probability. Let $k_{i}$ be the selected rectangle index, with orthonormal basis $n_{k_{i}}$, $u_{k_{i}}$, $v_{k_{i}}$. Binary indicators $\chi_{u,i}$ and $\chi_{v,i}$ equal one when the corresponding query coordinate is clamped at a finite bound. The dimensionless selected-feature projector is

(A23) $$P_{i}\left(T\right)=n_{k_{i}}n_{k_{i}}^{T}+\chi_{u,i}\left(T\right)u_{k_{i}}u_{k_{i}}^{T}+\chi_{v,i}\left(T\right)v_{k_{i}}v_{k_{i}}^{T},$$

Let $A_{i}$ be the Jacobian of the BIM-frame query with respect to a local six-dimensional pose increment, and let $j_{i}$ be the column gradient of its scalar distance. In the implementation, the rotational coordinates are length-scaled by the action metric, so all six increment coordinates are in meters and $A_{i}$ and $j_{i}$ are dimensionless. The equal-mass gradient g and positive-semidefinite Gauss–Newton/IRLS matrix H consequently have units m^−1^ and m^−2^, respectively:

(A24) $$g\left(T\right)=\frac{1}{N_{R}}\sum_{i=1}^{N_{R}}w_{i}\left(T\right)d_{i}\left(T\right)j_{i}\left(T\right),$$

(A25) $$H\left(T\right)=\frac{1}{N_{R}}\sum_{i=1}^{N_{R}}w_{i}\left(T\right)A_{i}{\left(T\right)}^{T}P_{i}\left(T\right)A_{i}\left(T\right)$$

### Appendix B.3. Action-Limited Update and Terminal Selection

Let VB be the complete set of unique finite-rectangle corners in BIM coordinates. *T* and *U* are rigid maps, each combining a dimensionless rotation and a translation in meters. Their physical change is measured by the corner-RMS action

(A26) $$d_{B}\left(T,U\right)=\sqrt{\frac{1}{\left|V_{B}\right|}\sum_{v\in V_{B}}∥Tv-Uv{∥}_{2}^{2}}$$

At each stage, a candidate update minimizes the local quadratic model within the current-pose chart. It is accepted only if its exact action satisfies the active 0.015, 0.0075, or 0.005 m bound (15, 7.5, or 5 mm) and the Armijo decrease test. The chart is re-anchored after every accepted update.

After equal refinement, the terminal risk uses a scale of 0.002 m, equivalent to 2 mm:

(A27) $$J_{2}\left(T\right)=\frac{1}{N_{R}}\sum_{i=1}^{N_{R}}\left[1-exp\left(-\frac{d_{i}{\left(T\right)}^{2}}{2{\left(0.002\right)}^{2}}\right)\right]$$

The deterministic selected pose is

(A28) $$\hat{m}=min\underset{1\leq m\leq M_{0}}{arg\;min}J_{2}\left(T_{F}^{\left(m\right)}\right),\;\;\hat{T}=T_{F}^{\left(\hat{m}\right)}$$

Let $A_{B}$ index the active paths in the nominal BIM seam set $C_{B}$, with $M_{g}$ ordered samples on class g. This calligraphic class set is distinct from the scalar surface area $A_{B}$ in Equation (5b); the synthetic evaluation uses only its two available flange paths. Each BIM-frame seam sample $s_{g,j}$ is transferred by

(A29) $${\hat{s}}_{g,j}=\hat{R}s_{g,j}+\hat{t},\;\;g\in A_{B},\;j=1,\;\ldots ,\;M_{g}$$

No initialization score, side information, numerical stopping state, or ground truth enters this terminal ordering.

## References

[1] Wang X., Yu H., McGee W., Menassa C.C., Kamat V.R. (2024). Enabling Building Information Model-driven human-robot collaborative construction workflows with closed-loop digital twins. Comput. Ind..

[2] Yoon S., Park M., Ahn C.R. (2025). Comparing dynamic viewpoint control techniques for teleoperated robotic welding in construction. Autom. Constr..

[3] Ahmed S.M., Tan Y.Z., Chew C.M., Al Mamun A., Wong F.S. Edge and corner detection for unorganized 3D point clouds with application to robotic welding. Proceedings of the 2018 IEEE/RSJ International Conference on Intelligent Robots and Systems (IROS).

[4] Takubo T., Miyake E., Ueno A., Kubo M. (2023). Welding line detection using point clouds from optimal shooting position. J. Robot. Mechatron..

[5] Wei P., Cheng S., Li D., Song R., Zhang Y., Zhang W. Coarse-to-fine detection of multiple seams for robotic welding. Proceedings of the 2024 IEEE/RSJ International Conference on Intelligent Robots and Systems (IROS).

[6] Huang S., Gojcic Z., Usvyatsov M., Wieser A., Schindler K. PREDATOR: Registration of 3D point clouds with low overlap. Proceedings of the IEEE/CVF Conference on Computer Vision and Pattern Recognition (CVPR).

[7] Mellado N., Aiger D., Mitra N.J. (2014). Super 4PCS: Fast Global Pointcloud Registration via Smart Indexing. Comput. Graph. Forum.

[8] Yang H., Shi J., Carlone L. (2021). TEASER: Fast and certifiable point cloud registration. IEEE Trans. Robot..

[9] American Institute of Steel Construction (2023). Steel Construction Manual.

[10] Peri S., Lee I., Kim C., Li F., Hermans T., Lee S. Point cloud models improve visual robustness in robotic learners. Proceedings of the 2024 IEEE International Conference on Robotics and Automation (ICRA).

[11] Dinham M., Fang G. (2013). Autonomous weld seam identification and localisation using eye-in-hand stereo vision for robotic arc welding. Robot. Comput. Integr. Manuf..

[12] Peng R., Navarro-Alarcon D., Wu V., Yang W. A point cloud-based method for automatic groove detection and trajectory generation of robotic arc welding tasks. Proceedings of the 2020 17th International Conference on Ubiquitous Robots (UR).

[13] Kim J., Lee J., Chung M., Shin Y.-G. (2021). Multiple weld seam extraction from RGB-depth images for automatic robotic welding via point cloud registration. Multimed. Tools Appl..

[14] Liu J., Jiao T., Li S., Wu Z., Chen Y.F. (2022). Automatic seam detection of welding robots using deep learning. Autom. Constr..

[15] Li Y., Xu Y., Wang X., Ma X., Wang Q., Zhang H. (2025). A feature-extraction localization algorithm research for teaching-free automated robotic welding based on 3D point cloud. Int. J. Adv. Manuf. Technol..

[16] Li T., Meng S., Lu C., Wu Y., Liu J. (2025). A novel BIM and vision-based robotic welding trajectory planning method for complex intersection curves. Measurement.

[17] Besl P.J., McKay N.D. (1992). A method for registration of 3-D shapes. IEEE Trans. Pattern Anal. Mach. Intell..

[18] Rusu R.B., Blodow N., Beetz M. Fast point feature histograms (FPFH) for 3D registration. Proceedings of the 2009 IEEE International Conference on Robotics and Automation (ICRA).

[19] Zhou Q.-Y., Park J., Koltun V., Leibe B., Matas J., Sebe N., Welling M. (2016). Fast global registration. Computer Vision—ECCV 2016.

[20] Qin Z., Yu H., Wang C., Guo Y., Peng Y., Ilic S., Hu D., Xu K. (2023). GeoTransformer: Fast and robust point cloud registration with geometric transformer. IEEE Trans. Pattern Anal. Mach. Intell..

[21] (2022). Code of Standard Practice for Steel Buildings and Bridges.

[22] Vince J. (2005). Geometry for Computer Graphics: Formulae, Examples and Proofs.

[23] Hoppe H., DeRose T., Duchamp T., McDonald J., Stuetzle W. Surface reconstruction from unorganized points. Proceedings of the 19th Annual Conference on Computer Graphics and Interactive Techniques (SIGGRAPH ‘92).

[24] Joo K., Oh T.-H., Kim J., Kweon I.S. Globally optimal Manhattan frame estimation in real-time. Proceedings of the IEEE Conference on Computer Vision and Pattern Recognition (CVPR).

[25] Barfoot T.D. (2017). State Estimation for Robotics.

[26] Holland P.W., Welsch R.E. (1977). Robust regression using iteratively reweighted least-squares. Commun. Stat. Theory Methods.

[27] Zhang J., Yao Y., Deng B. (2022). Fast and robust iterative closest point. IEEE Trans. Pattern Anal. Mach. Intell..

[28] Moré J.J., Sorensen D.C. (1983). Computing a trust region step. SIAM J. Sci. Stat. Comput..

[29] Armijo L. (1966). Minimization of functions having Lipschitz continuous first partial derivatives. Pac. J. Math..

[30] Hinterstoisser S., Lepetit V., Ilic S., Holzer S., Bradski G., Konolige K., Navab N., Lee K.M., Matsushita Y., Rehg J.M., Hu Z. (2013). Model based training, detection and pose estimation of texture-less 3D objects in heavily cluttered scenes. Computer Vision—ACCV 2012.

[31] Wilcoxon F. (1945). Individual comparisons by ranking methods. Biom. Bull..

